# The usefulness of serial ultrasound in thyroid mucosa-associated lymphoid tissue lymphoma

**DOI:** 10.3389/fendo.2022.1054584

**Published:** 2022-12-16

**Authors:** Xiumei Zhang, Boxiong Wei, Lin Nong, Hong Zhang, Ying Gao, Jinping Ou

**Affiliations:** ^1^ Department of Ultrasound, Peking University First Hospital, Beijing, China; ^2^ Department of Pathology, Peking University First Hospital, Beijing, China; ^3^ Department of Endocrinology, Peking University First Hospital, Beijing, China; ^4^ Department of Hematology, Peking University First Hospital, Beijing, China

**Keywords:** thyroid lymphoma, mucosa-associated lymphoid tissue (MALT), Hashimoto thyroiditis, ultrasound, thyroid imaging and reporting data system (TIRADS), follow-up

## Abstract

**Background:**

Mucosa-associated lymphoid tissue (MALT) lymphoma is an extranodal lymphoma with an indolent natural course. The thyroid gland is an uncommon site of involvement. We aimed to investigate serial ultrasound features and the disease progression during the clinical course of thyroid MALT lymphoma.

**Methods:**

We searched our hospital’s pathology database (5,418 patients with thyroid malignancy) between January 2000 and July 2022. The medical records and serial ultrasounds of 11 patients with 12 thyroid MALT lymphoma foci were analyzed retrospectively.

**Results:**

An enlarging neck mass, dyspnea, B symptoms, and neck lymphadenopathy were seen at diagnosis in 9 (9/11, 81.8%), 3 (3/11, 27.3%), 2 (2/11, 18.2%), and 9 (9/11, 81.8%) cases, respectively. Eleven cases were concomitant Hashimoto thyroiditis. Common ultrasound features included bilateral or unilateral asymmetric goiter or large, solid, and very hypoechoic nodules (11/12, 91.7%) interspersed with linear, reticular hyperechoic, and enhanced posterior echoes (11/12, 91.7%), and neck lymph node involvement (10/11, 90.9%). The Thyroid Imaging and Reporting Data System (TIRADS) categories showed higher diagnostic accuracy (11/12, 91.7%) than real-time ultrasound (2/12, 16.7%) in evaluating thyroid lesions for recommendation of fine-needle aspiration (FNA). Serial ultrasound showed self-limiting changes in three cases, relapse in three cases after subtotal thyroidectomy and chemotherapy, large cell transformation (LCT) in one case after left lobectomy, partial remission in one case, and complete remission after chemo/radiation in four cases; progression to enlarged thyroid nodules occurred in three cases without treatment, with no obvious change observed after diagnosis. Three patients died during follow-up.

**Conclusion:**

On sonograms, solid large thyroid nodules or goiter with very hypoechoic and enhanced posterior echoes in the setting of Hashimoto thyroiditis should raise suspicion for MALT lymphoma. TIRADS categories can improve the ultrasound diagnostic efficacy for malignancy. Serial ultrasound examinations demonstrated self-limiting and indolent natures of thyroid MALT lymphoma.

## Introduction

As the most common subtype of indolent lymphoma in China (1), mucosa-associated lymphoid tissue (MALT) lymphoma arises at any anatomical site. The thyroid is the exceedingly rare site of MALT lymphoma and affects 2% of all cases (2, 3). Thyroid ultrasound is the most helpful imaging modality to differentiate normal parenchyma from diffuse or nodular thyroid disease by evaluating thyroid size, echogenicity, echotexture, margins, and vascularity (4). Due to the scarcity of thyroid MALT lymphoma, few studies have been reported explicitly evaluating its ultrasound features during its clinical course. In addition, approximately 90% of thyroid lymphomas have evidence of Hashimoto thyroiditis, which precedes thyroid MALT lymphomas (5, 6). It is difficult for ultrasound radiologists to diagnose this disease early and distinguish it from Hashimoto thyroiditis due to the similar appearance of inflammation (7). The reports of real-time ultrasound cannot even identify patients who should be referred to further fine-needle aspiration (FNA), because thyroid lesions are often misdiagnosed as benign diseases due to interobserver differences in awareness of this disease.

The 2017 American College of Radiology-TIRADS (ACR-TIRADS) showed good performance in the diagnosis of thyroid nodules (TNs) (8). In 2020, the Chinese Medical Association proposed the Chinese-tirads (C-TIRADS), which is widely used in TNs evaluation (9). To reduce diagnostic discrepancy, we reviewed ultrasound features of thyroid MALT lymphoma and tried to use the 2017 ACR-TIRADS and 2020 C-TIRADS to assess this disease risk stratification to identify whether it warrants biopsy or ultrasound follow-up. However, neither guideline has been previously used to diagnose thyroid diffuse diseases. Serial ultrasound demonstrated thyroid MALT lymphoma’s natural course and response to treatment especially revealed that the disease could also stop growth, then decrease in size even disappear due to its self-limiting characteristic in the follow-up period. These findings can aid clinicians in obtaining a better understanding of the clinical presentations, ultrasound features, early diagnosis, optimized treatment management, and prognosis of thyroid MALT lymphoma.

## Methods

### Patients

The pathology database from January 2000 to July 2022 was reviewed, and 5,418 patients with thyroid malignancy and 37 patients with thyroid lymphoma were identified using the terms “thyroid”, “lymphoma”, “MALT”, “malignancy”, “carcinoma”, and “Hashimoto thyroiditis”. Eleven patients (three men and eight women) with MALT lymphoma were enrolled in this study. The median age was 63 years (range 58 to 80 years) at diagnosis. The inclusion criteria were as follows: ① pathologically proven thyroid MALT lymphoma by surgery or core-needle biopsy (CNB); ② thyroid ultrasound examinations were performed within 1–3 months at pathological diagnosis. Patient information was obtained from medical records and present condition was obtained by telephone follow-up.

### Ultrasound workup

Thyroid sonography scanning with neck hyperextension in a supine position was performed, and static images were stored in a picture archiving and communication system (PACS). The median follow-up for patients was 9 years (range 2 to 16 years). The ultrasound patterns of thyroid lymphoma were classified into three types: nodular, diffuse, and mixed types (10–12). The ultrasound features, including composition, echogenicity, shape/orientation, margin and echogenic foci, were evaluated by two ultrasound radiologists with more than 10 years of ultrasound experience in a consensus manner and uninformed pathological results. In addition to the above nonspecific features, certain characteristics suggesting thyroid lymphoma, such as internal echoes/echotexture and posterior echoes were also included. Malignancy-related ultrasound features consisting of hypoechoic/very hypoechoic echogenicity, taller-than-wide shape/vertical orientation, ill-defined or irregular margins (including extrathyroidal extension), punctate echogenic foci (PEF)/microcalcifications and solid were analyzed and used in diffuse thyroid disease in this study. Intravascularity and neck lymphadenopathy can help to distinguish the disorder nature and composition. The maximum diameter was measured in all sectional planes. Enlargement of the thyroid was identified as an anteroposterior diameter of the thyroid > 2 cm. Serial previous and follow-up ultrasound examinations were also reviewed to gain substantial insight into the clinical course of thyroid MALT lymphoma over time. Abnormal appearances of neck lymph nodes include a globular shape or diameter of the short axis > 5 mm, loss of the normal echogenic hilum, presence of peripheral rather than hilar flow or chaotic vascularity, heterogeneity with cystic components, and PEF (8, 9). Any neck lymph node with the above suspicious features or very hypoechoic and enhanced posterior echo was considered to be involved in MALT lymphoma. The real-time ultrasound diagnosis was recorded.

### 2017 ACR-TIRADS and 2020 C-TIRADS analysis

The ultrasound features of TNs were assigned points and categorized based on the 2017 ACR-TIRADS and 2020 C-TIRADS (8, 9). The margin of diffuse diseases was identified as follows: The goiter or enlarged gland filled with hypoechoic/very hypoechoic was defined as irregular and assigned 2 points. The presence of border abutment, contour bulging, trachea, and esophagus wrapped by disorder is defined as extrathyroidal extension, assigned 3 points in ACR-TIRADS. Above all assigned 1 point respectively in the C-TIRADS. FNA or ultrasound follow-up was recommended based on the risk level of the two guidelines and its maximum diameter.

### Statistical analysis

Continuous data of normal distribution (age, size, and resistive index) were described as mean ± standard deviation, and categorized data (gender, some ultrasound features, ACR- and C-TIRADS grade, etc.) were described as percentages. SPSS 24.0 software (IBM, Armonk, NY) was used for statistical analysis.

## Results

### Clinical features

All 11 patients had a 6-month to 10-year history of goiter, TNs, thyroid diffuse diseases, increased thyroid antibody and Hashimoto thyroiditis (over 10-year history in two cases) before diagnosis. Three patients had previous overt hypothyroidism and received levothyroxine. Eleven cases were pathologically diagnosed with concomitant Hashimoto thyroiditis. The most common symptom was a gradually or rapidly (within 1–6 months) growing neck mass or goiter in nine cases (9/11, 81.8%) and was followed by dyspnea (3/11, 27.3%) and accompanied B symptoms (2/11, 18.2%). Eleven patients presented with stage IE (1/11, 9.1%), stage IIE (6/11, 54.6%), stage IIIE (2/11, 18.2%), and IVE (2/11, 18.2%). Two cases (2/11, 18.2%) involved multiple extranodal sites constituting the stomach and intestine. Generalized nodal involvement was rare and only seen in one case (1/11, 9%). One patient had previously undergone intestinal excision and was diagnosed with MALT lymphoma 7 years before, and one case had been proved to be marginal zone lymphoma (MZL) of the lymph node by CNB 6 years before. One patient simultaneously involved thyroid and gastric, which was diagnosed pathologically by CNB and gastroscopy.

### Ultrasound and TIRADS categories and recommendations

**Figure 1:**
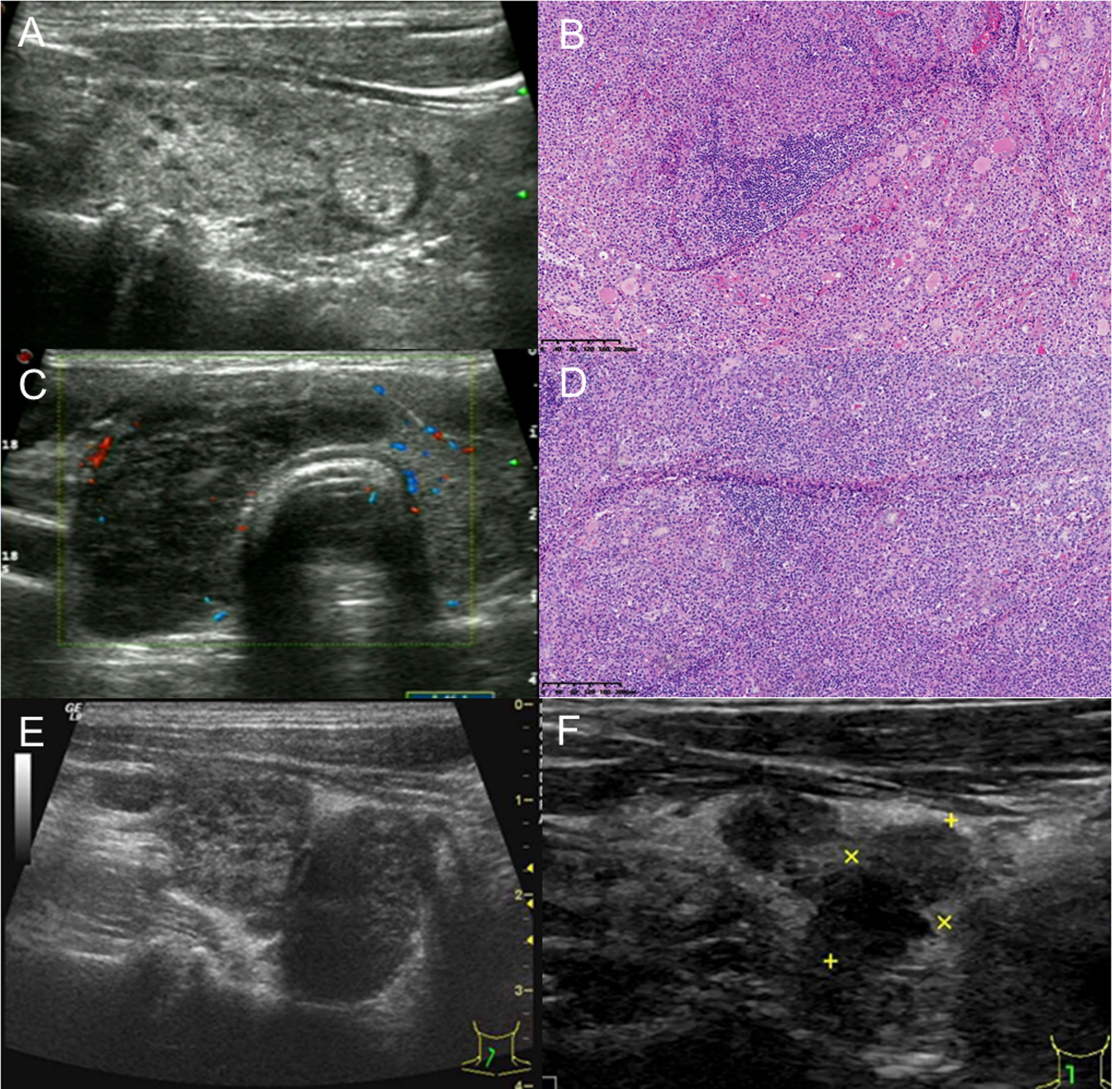
A 61-year-old man with thyroid MALT lymphoma underwent subtotal thyroidectomy. **(A)** Left thyroid with MALT lymphoma and concomitant Hashimoto thyroiditis: a rare ultrasonic manifestation of diffuse micronodules. **(B)** The left thyroid parenchyma was effaced by a sheet-like and nodular infiltrate of small- to medium-sized lymphoid cells with centrocyte-like, monocytoid, and plasmacytoid appearance. Lymphoepithelial lesions and some residual follicles were noted (H&E, ×100). **(C)** The markedly enlarged right thyroid with very hypoechoic misdiagnosed as Hashimoto thyroiditis on the real-time ultrasound report. It was categorized as TR5 with 7 points based on the 2017 ACR-TIRADS, 4C with 3 points based on the 2020 C-TIRADS, and FNA was recommended. **(D)** The right thyroid follicles and parenchyma were eventually effaced by diffuse infiltrate of atypical lymphoid cells throughout the lobe, and cytological features resembled the left thyroid. Several strands interspersed within the tumor cells implied interstitial fibrosis (H&E, ×100). **(E)** Ultrasound showed a rapidly enlarged residual right thyroid gland presenting as two adjacent very hypoechoic solid masses at 6 weeks after surgery. **(F)** After another 8 years, the two masses decreased in size with no treatment.

Thyroid MALT lymphoma was classified into three patterns on sonograms (**Table 1**). The diffuse type was the most common pattern (5/11, 45.5%), and nodular and mixed types appeared in an equal proportion (3/11, 27.3%) of cases. MALT lymphoma presented with enlarged solid nodules and goiter with very hypoechoic, enhanced posterior echoes and almost increased vascularity relative to the normal thyroid parenchyma at diagnosis. There were variable internal hyperechoic echoes, such as linear, reticular, and alveolate, as well as an absence of calcification. Only one exception in one case showed different ultrasonic findings from the above description, presented with multiple hypo/isoechoic micronodules (3–10 mm) interspersed within parenchyma of normal size of left thyroid with MALT lymphoma, and was proved by excisional specimen (**Figure 1**). Two cases (2/11, 18.18%) demonstrated deviation and stenosis of the trachea. Ultrasound revealed neck lymph node involvement in 10 cases (10/11, 90.9%), with very hypoechoic, enhanced posterior echoes, rich vascularity, and a short axis > 5 mm.

**Table 1:** The size of thyroid MALT lymphoma in three ultrasound patterns at diagnosis (*n* = 11).

Ultrasound pattern	The anteroposterior diameter of thyroid (cm, mean ± SD)		
Left lobe	Right lobe	Isthmus
Diffuse type (*n* = 5)	3.8 ± 1.3	3.5 ± 1.4	2.2 ± 0.6
Nodular type (*n* = 3)	3.2 ± 0.8	2.5 ± 0.7	1.1 ± 0.9
Mixed type (*n* = 3)	1.9 ± 0	2.7 ± 0.7	0.7 ± 0.6
Total	3.2 ± 1.2	3.0 ± 1.1	1.5 ± 0.8
Maximum diameter of TNs with MALT lymphoma	5.1 ± 1.7

Based on the ultrasound features, the 2017 ACR-TIRADS and 2020 C-TIRADS showed similar diagnostic performance for thyroid MALT lymphoma. The excellent diagnostic efficacy of the two guidelines was higher than that of real-time ultrasound. The one exception was a case in which left thyroid with MALT lymphoma was misdiagnosed as mildly suspicious and benign disease on the basis of the ACR-TIRADS and C-TIRADS and did not meet the criteria for FNA. All details mentioned above are summarized in **Table 2**.

**Table 2:** Demographic, clinical, and ultrasound features of thyroid MALT lymphoma.

Patients	*n* = 11
Age, mean ± SD, years	65.73 ± 7.31
Gender	
Male (%)	3 (27.3%)
Female (%)	8 (72.7%)
Chief symptoms	
Neck mass enlarged	9 (81.8%)
Stomach discomfort	1 (9.1%)
Dyspnea	3 (27.3%)
Accompanied B symptoms	2 (18.2%)
Concomitant Hashimoto thyroiditis	11 (100%)
Tumor histology	
Intestine MALT lymphoma	1 (9.1%)
Lymphocytic leukemia	1 (9.1%)
lymph nodes MZL (Peripheral)	1 (9.1%)
Myoma of uterus	1 (9.1%)
Other site involvement	
Gastric MALT lymphoma	1 (9.1%)
Intestine MALT lymphoma	1 (9.1%
Bone involvement	1 (9.1%)
Systemic dissemination	1 (9.1%)
Other tumor and disease	
Lymphocytic leukemia	1 (9.1%)
Secondary myelofibrosis	1 (9.1%
Lung cancer	1 (9.1%)
Ann Arbor Staging for thyroid MALT lymphoma(Location of Disease Outside of the Thyroid)	
IE	1 (9.1%)
IIE	6 (54.6%)
IIIE	2 (18.2%)
IVE	2 (18.2%)
Large B-cell transformation	1 (9.1%)
Neck lymphadenopathy (%)	
Yes	10 (90.9%)
No	1 (9.1%)
Size of short axis, mean ± SD, mm	9.0 ± 2.9
Ultrasound features (Based on ACR-TIRADS and C-TIRADS)	*n* =12^a^
Composition	
Cystic or almost	0 (0.0%)
Solid	12 (100.0%)
Echogenicity	
Hypoechoic	1 (8.3%)
Very hypoechoic	11 (91.7%)
Shape	
Taller-than-wide	8 (66.7%)
Wider-than-tall	4 (33.3%)
Margin	
Ill-defined	2 (18.2%)
Irregular or spiculated or lobulated	3 (27.3%)
Extrathyroidal extension	6 (54.5%)
Echogenic foci	
Calcification	0
Punctate of undetermined significance	1 (8.3%)
Variable internal echogenic^b^	
Linear	6 (50.0%)
Reticular/alveolate	7 (58.3%)
Irregular stripe/cord-like	6 (50.0%)
Posterior echo	
Enhancement	11 (91.7%)
No effect	1 (8.3%)
Increased vascularity (%)	12 (100.0%)
Pulsed wave imaging (*n* = 5)^c^	
Resistive index, mean ± SD	0.66 ± 0.15
Peak systolic velocity, mean ± SD, cm/s	23.32 ± 10.06
Recommendation by real-time ultrasound	
Biopsy	5 (41.7%)
Follow-up	3 (25.0%)
Not available	4 (33.3%)
ACR-TIRADS risk levels	
TR3 for 5%	1 (8.3%)
TR4 for 5–20%	4 (33.3%)
TR5 for at least 20%	7 (58.4%)
C-TIRADS category for malignancy risk rate (%)	
4a for 2–10%	1 (8.3%)
4b for 10–50%	0
4c for 50–90%;	9 (75.0%)
5 > 90%	2 (16.7%)
Recommendation by ACR TI-RADS (%)	
FNA	11 (91.7%)
Follow-up	1 (8.3%)
Recommendation by C-TIRADS (%)	
FNA	11 (91.7%)
Follow-up	1 (8.3%)

a Discrepant ultrasound manifestations of the left and right thyroid glands were evaluated respectively in the setting of MALT lymphoma involving both lobes in one case. Therefore, the total number of thyroid MALT lymphoma foci was 12.

b Multiple kinds of echogenicity in one lesion, so the total exceeded 11.

c Only five cases with PW.

### Serial ultrasound during the clinical course and treatment management

**Figure 2:**
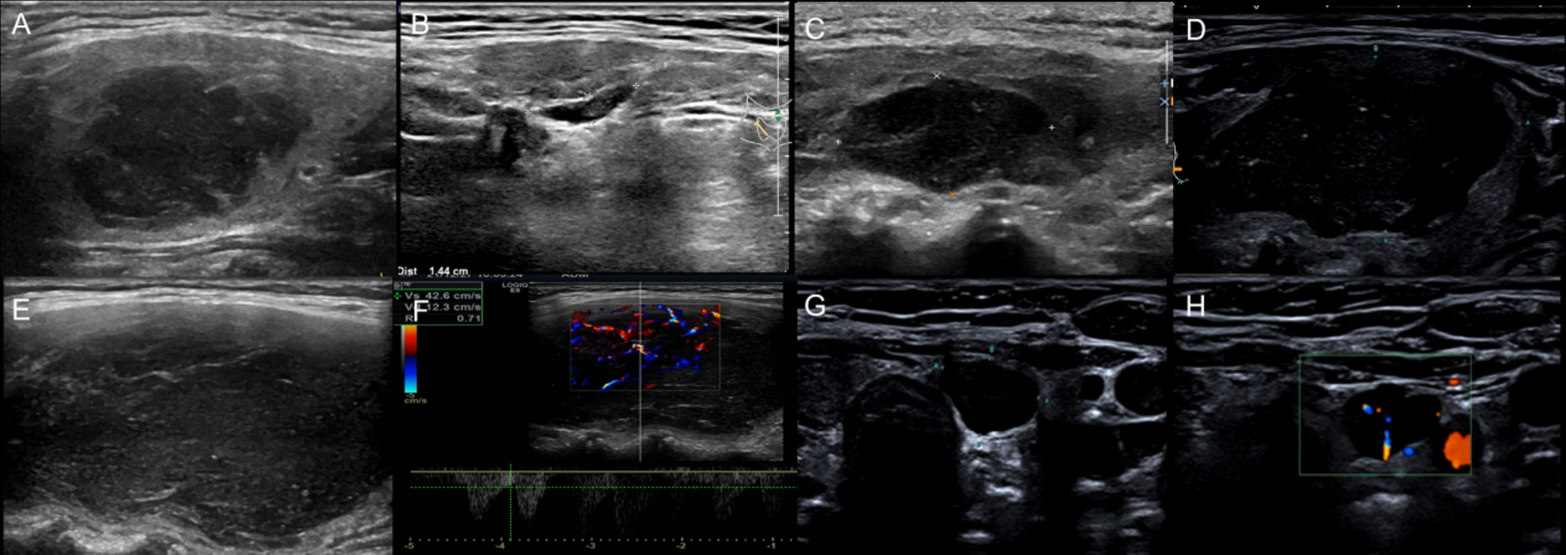
Serial US demonstrated changes in the clinical course of MALT lymphoma in a 67-year-old woman with nodular lymphoma. **(A)** A 3.3× 2.1 cm very hypoechoic mass of the right thyroid. **(B)** The mass reduced to 1.4 × 0.4 cm without treatment after 3 months. **(C)** After 2 years and 5 months, a 2.2 × 1.4 cm mass of the left thyroid was found. **(D)** Six months later, the mass of the left thyroid had obviously enlarged to 4.7×2.2 cm with very low internal echogenicity, and the diagnostician at the time thought it was a cystic nodule with hemorrhage. **(E)** After another 2 months, the mass enlarged to 5.0 × 2.8 cm, and the lymph nodes also enlarged. **(F)** One month later, the mass had grown up to 6.0 cm and was rich in blood flow with an RI of 0.7. **(G)** Another “cystic” nodule with enhanced posterior echo. **(H)** The “cystic” nodule had a central blood flow and was diagnosed as a solid nodule.

Serial ultrasound showed the natural disease course of thyroid MALT lymphoma and its response to the treatment. The indolent, self-limiting natures of thyroid MALT lymphoma were found and are illustrated in **Figures 1**, **2** and **Table S1**. Details are as follows: ① Three patients did not receive any treatment management and were under active surveillance by ultrasound examination for 4, 9, and 10 years. The first patient was a 67-year-old woman. Serial ultrasound showed that a very hypoechoic nodule of the right thyroid disappeared 6 months after being detected. Then, a similar nodule of the left thyroid appeared and progressively grew and occupied almost the whole thyroid, changing from the nodular to mixed type over 3 years (**Figure 2**). After 6 months, the left thyroid tumor rapidly decreased in size from 6.0 to 2.0 cm. The second case, a 66-year-old woman with at least a 5-year history of Hashimoto thyroiditis, developed very hypoechoic nodules of the right thyroid that gradually grew up to become the diffuse type with markedly enlarged goiter. The third case, a 75-year-old woman, had undergone 16 times thyroid ultrasound workups. One very hypoechoic nodule appeared in the isthmus; went through a process of enlargement, shrinkage, and disappearance; and then appeared again during the 10-year follow-up. The thyroid gland and neck lymph nodes also increased and decreased in size accordingly at the same time. ② Three patients underwent debulking surgery. Localized relapses with rapidly enlarged residual glands detected by ultrasound at 4 and 6 weeks after subtotal thyroidectomy in two cases. One patient did not receive any additional treatment subsequently, and the enlarged residual gland had reduced in size after 8 years (**Figure 1**). The patient died of multiple adenocarcinomas in the right lung 9 years later. Another patient underwent excision of the residual tumor at 6 weeks after the first surgery, survived for 1.5 years, and was disease free to date. In the third case, large cell transformation (LCT) occurred in the contralateral (right) peripheral, abdominal, and retroperitoneal lymphadenopathy at 1.5 years after left lobectomy and was confirmed by CNB. ③ Two cases received local thyroid radiotherapy and obtained a better response. A 63-year-old man with IIE stage and a history of excisional intestinal MALT lymphoma 7 years before survived for 12 years and was relapse free. Another 80-year-old woman with IIE stage survived for 2 years with disease-specific to date after radiotherapy, and ultrasound also showed thyroid size and echo returned to normal. ④ Normal thyroid size and hypo/isoechoic parenchymal echogenicity were obtained in three cases after chemotherapy. One of them, a 58-year-old woman with concomitant gastric and thyroid MALT lymphoma developed systemic dissemination, died of brain involvement and hemorrhage after 2.5 years. The other one, a 59-year-old woman with a 7-year history of MZL, concomitant lymphocytic leukemia and bone marrow involvement, survived for 9 years after chemotherapy, and then died of lung infection. The third case achieved complete remission for 7 years and relapse with goiter detected by ultrasound.

## Discussion

Thyroid MALT lymphoma is extremely rare, accounting for 0.2% (11/5,418) of thyroid malignancies and 29.7% (11/37) of thyroid lymphomas, within the range of 6–46.8% reported in some studies (5, 6, 13). Among 11 cases with thyroid MALT lymphoma, 72.7% (8/11) occurred in women, 81.8% (9/11) were older than 60 years, and 100% (11/11) arose in a background of Hashimoto thyroiditis, which are consistent with previous studies (3, 14). There are thyroid-specific sex differences with a female predominance in this study. Most patients with thyroid MALT lymphoma occur in adults, with a median age in the seventh decade of life. Hashimoto thyroiditis, that is, a form of autoimmune-based chronic inflammation, is known to precede thyroid MALT lymphomas. In patients with Hashimoto thyroiditis, the risk of thyroid lymphoma is 70 times that in the general population, and approximately 85% are MALT lymphomas. Hashimoto thyroiditis is very common, but thyroid lymphoma is very rare, although it is associated with Hashimoto thyroiditis. It usually takes approximately 3–18 years to evolve into MALT lymphoma (15, 16). The median age of patients with Hashimoto thyroiditis, 40–55 years, is younger than that of patients with thyroid MALT lymphoma (17). Many MALT lymphoma cases have a history of chronic inflammatory disorder that results in the accumulation of extranodal lymphoid tissue (called acquired MALT). One postulated mechanism was a slow malignant transformation from chronic antigenic stimulation of marginal lymphocytes (18).

Thyroid MALT lymphoma commonly presents as a rapidly or gradually enlarged neck mass and goiter leading to local compressive symptoms. This neck mass rapidly grows in a short time, which is rarely observed in Hashimoto thyroiditis (19). Dyspnea was the most common compressive symptom, presented in three cases (27.3%) due to tracheal stricture or deviation secondary to the diffusely enlarged thyroid lymphoma. The remaining symptoms, such as neck pain, dysphagia, and hoarseness, were not present in our cases. Classic B-type symptoms occurred in two cases (18.2%) with stage IVE disease, similar to that reported in approximately 20% of patients (20). Most patients presented with stage I or II disease (7/11, 63.8%). Stage IIIE and IVE disease were seen in 36.4% (18.2%, respectively) of cases, higher than the incidence of 2–7% of cases with primary thyroid lymphoma (5, 10), and similar to the incidence of 23–40% of cases with thyroid MALT lymphoma involving multiple extranodal sites (14, 21). Staging in patients with multiple extranodal lesions may be challenging because at least some cases with multiple site lesions perhaps constitute multiple clonally unrelated proliferations rather than truly disseminated disease (22).

Most thyroid MALT lymphoma presented as very hypoechoic solid enlarged nodules and/or goiter of tumor cell infiltration with enhanced posterior echoes on thyroid sonogram. Thyroid MALT lymphoma is composed of small B cells, including marginal zone cells with a prominent population of small and intermediate-sized lymphoid cells, accompanied by lymphoepithelial lesions and/or plasmacytic differentiation in some cases. The consistency of the tumor cells without any acoustic impedance results in enhanced posterior echoes. The rarest heterogeneous appearance of thyroid MALT lymphoma closely resembled Hashimoto thyroiditis, with multiple hypo/isoechoic micronodules (≤ 10 mm) interspersed within a normal sized left lobe gland. The pathological image and the sonogram showed the same size of the foci tumor cell infiltration. This is the first study to characterize the diffuse micronodular appearance of thyroid MALT lymphoma in the background of Hashimoto thyroiditis with normal-size gland. Diffuse micronodular disease with MALT lymphoma cannot be identified except with surgical pathology. Furthermore, from the dynamic perspective, the tumor developing course is the process of small single nodule or multiple tumor foci grow up to be nodular type, then progress to become mixed type and finally infiltrate throughout the thyroid to develop into diffuse type based on these patients’ serial ultrasound findings.

Serial ultrasound showed variable internal echoes of thyroid MALT lymphomas. The internal echoes were very low hypoechoic (i.e., pseudocystic), PEF of undetermined significance, and short linear, parallel linear, reticular/alveolate hyperechoic, sequentially appeared, and increased as the tumor grew up. Due to the heterogeneous appearance of thyroid MALT lymphomas on sonograms, differentiation of these lymphomas from benign diseases such as Hashimoto thyroiditis, goiter, hemorrhage in adenoma, and even cysts is difficult for radiologists with insufficient awareness of thyroid MALT lymphoma. In some cases with the nodular type, pseudocystic appearance or concomitant PEF of undetermined significance were often misdiagnosed as cyst or hemorrhage. Increased vascularity detected by color Doppler flow imaging (CDFI) and pulsed wave (PW) can be helpful to identify the pseudocystic nodule as solid. Approximately 90% of MALT lymphoma cases demonstrated increased disordered vascularity.

In our study, only two cases of suspected lymphoma were reported by two radiologists with more than 10 years of experience. Therefore, there was a marked discrepancy in the clinical real-time ultrasound reports. Application of the 2017 ACR-TIRADS or 2020 C-TIRADS categories is very useful to address ultrasound diagnostic dilemmas and eliminate interobserver differences in terms of recommendations for FNA/CNB. Because CNB can yield a higher proportion of diagnostic results than conventional FNA (23), our patients underwent CNB instead of FNA. Eleven abnormalities (11/12, 91.67%) detected in two TIRADSs were recommended for FNA/CNB. The one exception with the largest nodule ≤ 10 mm (**Figure 1**), which was categorized as TR3 and 4A nodules in ACR-TIRADS and C-TIRADS, was missed because the nodule size did not meet the recommended criteria for FNA. In such cases, active surveillance by follow-up ultrasound can substantially mitigate the possibility that significant malignancies remain undetected over time. Once the tumor size increases by more than 20% (24), biopsy is necessary for a precise diagnosis of the subtype, which affects subsequent therapeutic management. In addition, patients with debulking surgery should receive active surveillance by follow-up ultrasound to detect early local recurrence. Ultrasound revealed excisional tumor-infiltrated thyroid capsules in one case. The presence of extrathyroidal extension and abnormal lymph nodes increase the risk stratification of disease in TIRADS clinical practice (9). Nevertheless, in our study, neck lymphadenopathy involvement was found in up to 90.9% (10/11) of the patients at diagnosis, possibly because most of these patients did not decide to perform biopsies until the presence of apparently enlarging neck masses.

MALT lymphomas have an indolent natural course and are slow to disseminate. Because of their insidious onset, precise diagnosis of thyroid MALT lymphomas is usually obtained after a long time (1–10 years) from the initial visit. In this study, serial sonograms showed the complicated natural course of thyroid MALT lymphoma, that is, thyroid MALT lymphoma behaved alternating or sequential self-limiting, progression or being unchanged on serial ultrasound for many years (25). This self-limiting property of thyroid MALT lymphoma is likely similar to some juvenile thyroid cancer and papillary thyroid microcarcinoma (PTM) in the patients > 40 years old (26).

With regards to treatment strategy, MALT lymphomas are sensitive and have a better response to local thyroid radiotherapy, which is followed by prolonged disease-free intervals. Early stage MALT lymphomas may be treated with radiotherapy alone without disease recurrence. The follow-up ultrasound showed thyroid reduced to normal size without the nodule in two cases after radiotherapy. Because radiotherapy may result in severe adverse events, observation or rituximab alone is also used. Stages III and IV refer to the treatment of rituximab combined with chemotherapy or observation (1). The most common histologic subtype of thyroid lymphoma is diffuse large B-cell lymphoma (DLBCL) which accounts for 43.3–70% of cases. And LCT or mixed MALT/DLBCL accounts for approximately 4–7.6% (5, 27). In patients with transformation into DLBCL, mixed subtypes, and/or extensive bulky local disease, multimodal treatment with rituximab, combination chemotherapy, and local radiotherapy provides the highest overall survival rates. One patient with stage IE disease (1/11, 9.1%) developed LCT in the contralateral half of the systemic lymphadenopathy at 1.5 years after left lobectomy and combined chemotherapy to achieve complete remission in the study. The prognosis for MALT lymphoma is excellent except for transformation into DLBCL with the clinical behavior being more similar to DLBCL (reported in < 10% of cases) (28, 29).

The role of surgical intervention remains controversial, and such intervention is typically incidental in the management of indolent MALT lymphomas for other indications, such as palliation in the setting of critical airway obstruction or stenosis, which can be replaced by corticosteroids and chemo/radiotherapy and temporary use of tracheal stent (30). Among the three cases with debulking surgery, two cases (2/3, 66.7%) relapsed within a short time and one case developed aggressive LCT, then one case even developed second cancer (lung cancer). These unfavorable outcomes may be associated with autoimmune condition including persistence of Hashimoto thyroiditis. It raises a question of whether total thyroidectomy is a matter of better therapeutic efficacy due to absence of inflammatory stimuli of thyroiditis. Therefore, we propose that total thyroidectomy instead of debulking surgery is used in the clinical management in order to eradicate the strong risk factor of inflammation in Hashimoto thyroiditis. Regional radiotherapy and total thyroidectomy can avoid residual tumor relapse, LCT, and dissemination to multiple sites. However, the clinical benefits of extensive surgery are also controversial and need to be confirmed in large studies (6, 19, 31). Some experts believe that surgery is contraindicated for the patients with thyroid MALT lymphomas higher than stage IE, bulky tumors greater than 10 cm in size, and mixed tumors.

In this study, the goiter reduced to a normal size and returned to hypo/isoechoic parenchyma after chemotherapy in three cases, complete remission was 100%. Two cases went through a relative long period of remission (8 and 9 years); one case died and another relapsed. The third case with systemic dissemination had a 6-year history of goiter, died of brain involvement and hemorrhage after 2.5 years. Combined chemotherapy for advanced-stage disease can achieve complete remission in 90–100% of patients. However, relapses are unfortunately common within 3–6 years (10–35%), which can occur after many years and may involve other extranodal sites. The 59-year-old woman with a 7-year history of MZL, concomitant lymphocytic leukemia and bone marrow involvement, survived for 9 years after chemotherapy and then died of lung infection. In summary, mortality (3/11, 27.3%) was unfavorable; survival to date was 8.5 years (2–16 years) in our study.

In addition, three cases without treatment experienced a period of time (6 months, 2 years, and 3 years) with no significant change of tumor, survived for many years (4, 9, and 10 years) with disease-specific to date. We found thyroid MALT lymphomas disappeared, appeared, increased, shrunk, and even reoccurred on serial sonograms in two cases without treatment. Relapsed masses decreased in size in one case at 8 years after subtotal thyroidectomy during a long period of follow-up ultrasound. It is first time that the self-limiting changes of thyroid MALT lymphoma was demonstrated in the form of ultrasonic images, which has not been reported in the literatures we read. Thyroid MALT lymphomas in the above three cases (3/11, 27.3%) with self-limiting change showed paradoxical and complicated growing features. Although self-limiting growth seemed to show an excellent prognosis, it is difficult for MALT lymphoma to identify its growth arrest because it could restart proliferating and rapidly enlarging up to spread into diffuse goiter in the case of occurrence of self-limiting. The evolution is a bit different from those juvenile thyroid cancer and PTM (26). It is yet uncertain whether to progress to cancer death in the setting of two of three cases are alive to date; the third one died of another cancer of lung adenocarcinoma. The answer of this question is bestowed in the future maybe after many years, and this self-limiting mechanism needs further investigation in more profound level.

Thyroid MALT lymphoma follows a relatively benign course and historically demonstrates a better response to treatment. The 5-year disease-free survival rates approach 96–100%, higher than 71–75% for DLBCL and the other histological subtypes (19, 32). Poor prognostic factors include advanced age and stage, the presence of DLBCL, lack of treatment with radiation, greater tumor size, mediastinal involvement, rapid clinical growth, and the presence of B symptoms. However, there is the opposite opinion that involvement of multiple extranodal sites and even bone marrow involvement do not appear to confer a worse prognosis (28, 29). In the end, the ideal treatment management should remain relative indolent and self-limiting conditions for many years and preserve the patients’ quality of life and longevity.

There are some shortcomings in the present study. Surgical excision in three cases had superior diagnostic accuracy in comparison with CNB in eight cases. Biopsied tissue samples do not represent pathological features of entire tumor involvement with insufficient evidence, affecting the analysis of ultrasound characteristics. The other major limitation was the small sample size due to the morbidity and longer clinical course of thyroid MALT lymphoma, which failed to reach statistical significance. Further studies with large cohorts of patients and comprehensive analysis are needed to address this issue.

## Conclusions

Thyroid MALT lymphoma demonstrates an indolent natural course and a self-limiting trend. Most patients have an excellent prognosis. Thyroid ultrasound is the most sensitive method to detect thyroid MALT lymphoma. Enlarged very hypoechoic solid TNs or goiter with enhanced posterior echoes in the setting of Hashimoto thyroiditis on sonogram should raise suspicion for thyroid MALT lymphoma. Both the ACR-TIRADS and C-TIRADS categories can significantly improve ultrasound diagnostic efficacy to recommend FNA/CNB and decrease interobserver diagnostic discrepancy in clinical management. Serial ultrasound is helpful for better understanding of the indolent clinical course, self-limiting, relatively benign or aggressive behavior such as LCT, and better response to treatment management in thyroid MALT lymphoma. The TN risk stratification is also applicable to thyroid diffuse diseases. Active surveillance by the follow-up ultrasound TIRADS category can substantially screen significant malignancies.

## Data availability statement

The raw data supporting the conclusions of this article will be made available by the authors, without undue reservation.

## Author contributions

XZ contributed to the conception and design of this study. Material preparation and data collection were performed by XZ, BW, LN, HZ, YG, and JO. Analysis was performed by XZ and BW. The first draft of the manuscript was written by XZ and BW. All authors commented on previous versions of the manuscript and read and approved the final manuscript.
