# Supplementary material for: The usefulness of serial ultrasound in thyroid mucosa-associated lymphoid tissue lymphoma
Source: Front Endocrinol (Lausanne). 2022 Dec 16;13:1054584. doi: 10.3389/fendo.2022.1054584 (PMC9802905; doi:10.3389/fendo.2022.1054584)
Supplement: Supplementary file 1 [file Table_1.docx]

| **Table S1.** Serial ultrasound findings during the clinical course and treatment management of thyroid MALT lymphoma | | | | | | | |
| --- | --- | --- | --- | --- | --- | --- | --- |
| case | Age/sex | Tumor | Treatment | US pattern | Serial US finding | follow-up | Clinical course |
| 1 | 67/female | Right thyroid MALToma*  left thyroid MALToma | No treatment | Nodular type  Mixed type | The TN shrunk and disappeared  A left TN appeared, spread into  most of thyroid, then shrunk | After 3-6 months  During 3 years  Survival for 4 years | Self-limiting  Progression |
| 2 | 66/female | Right thyroid MALTomas | No treatment | Nodular type  Diffuse type | The TNs enlarged and gradually spread into the whole thyroid | During 9 years | Progression |
| 3 | 75/female | Thyroid MALToma  Neck lymph nodes | No treatment | Mixed type | Two TNs enlarged, shrunk, disappeared, reappeared  Ditto | During 10 years  (16 US examinations) | Self-limiting  progression |
| 4 | 60/male | Left thyroid MALToma | left lobectomy | Nodular type | No TN | During 1.5 years | Disease-free |
|  |  | Lymph nodes LCT*  (Right peripheral,  abdominal, and  Retroperitoneal) | No treatment |  | Multiple lymph nodes enlarged | 1.5-2 years after  left lobectomy | Progression |
|  |  |  | Chemotherapy |  | Neck lymph nodes enlarged | After 4 years | Part remission |
| 5 | 61/female | thyroid MALToma  Right residual masses | No treatment  Subtotal thyroidectomy  resection of masses | Mixed type  Diffuse type | The tumor enlarged and  spread into the whole thyroid  Residual right thyroid enlarged  No neck mass | 3.5 years before surgery  2-4 weeks after surgery  3 months later  Survival for 7.5 years | Progression  Relapse  Disease-free |
| 6 | 61/male | Thyroid MALToma  Right residual masses | Subtotal thyroidectomy  No treatment | Mixed type | Residual right thyroid enlarged  The masses decreased in size | After 4 and 6 weeks  After 8 years | Relapse  Self-limiting |
|  |  | Right pulmonary cancer | Surgery + Chemotherapy |  |  | After 9 years | death |
| 7 | 63/male | Intestinal MALToma | Surgery |  |  | 7 years before | Relapse-free |
|  |  | Thyroid MALToma | Radiotherapy | Nodular type | Normal size and echo | Survival for 12 years | Relapse-free |
| 8 | 80/female | Thyroid MALToma | Radiotherapy | Diffuse type | Normal size and echo | Survival for 2 years | Relapse-free |

continue to the next page

| 9 | 58/female | Thyroid MALToma  Gastric MALToma  Intestinal MALToma  Systemic disseminated | Chemotherapy | Diffuse type | Thyroid with normal size | A 6 years history of goiter | Remission |
| --- | --- | --- | --- | --- | --- | --- | --- |
|  |  | Brain MALToma and hemorrhage |  |  |  | 2.5 years later | death |
| 10 | 59/female | lymph nodes MZL* (Peripheral) | Chemotherapy |  | lymph nodes enlarged  normal lymph nodes | 7 years before | Remission |
|  |  | Thyroid MALToma  Lymphocytic leukemia  Secondary myelofibrosis  Bone involvement | Chemotherapy | Mixed type | Normal thyroid（size and echo） | 9 months later  9 years later | Remission  Death from lung infection |
| 11 | 73/female | Thyroid MALToma | Chemotherapy | Diffuse type | Normal thyroid（size and echo） | 10 months later | Remission |
|  |  |  | No treatment | Mixed type | The goiter with heterogeneous  hypoechoic | 7 years later  Survival for 8 years | Relapse |
| *MALToma: MALT lymphoma. *MZL: Marginal Zone Lymphoma. *LCT: large cell transformation | | | | | | | |
